# Deep‐targeted sequencing of endothelial nitric oxide synthase gene exons uncovers exercise intensity and ethnicity‐dependent associations with post‐exercise hypotension

**DOI:** 10.14814/phy2.13510

**Published:** 2017-11-28

**Authors:** Linda S. Pescatello, Elizabeth D. Schifano, Garrett I. Ash, Gregory A. Panza, Lauren M. L. Corso, Ming‐Hui Chen, Ved Deshpande, Amanda Zaleski, Burak Cilhoroz, Paulo Farinatti, Beth A. Taylor, Rachel J. O'Neill, Paul D. Thompson

**Affiliations:** ^1^ Department of Kinesiology University of Connecticut Storrs Connecticut; ^2^ Institute for Systems Genomics University of Connecticut Storrs Connecticut; ^3^ Department of Statistics University of Connecticut Storrs Connecticut; ^4^ School of Nursing Yale University New Haven Connecticut; ^5^ Department of Preventive Cardiology Hartford Hospital Hartford Connecticut; ^6^ Department of Physical Activity Sciences Rio de Janeiro State University Rio de Janeiro Brazil; ^7^ Department of Molecular and Cell Biology University of Connecticut Storrs Connecticut

**Keywords:** Blood Pressure, Exercise, Hypertension, Polymorphism

## Abstract

In previous studies, we found an endothelial nitric oxide synthase gene (*NOS3*) variant rs2070744 associated with the ambulatory blood pressure (BP) response following bouts of moderate and vigorous intensity acute exercise, termed post‐exercise hypotension (PEH). In a validation cohort, we sequenced *NOS3* exons for associations with PEH. Obese (30.9 ± 3.6 kg^.^m^−2^) African American (*n* = 14) [AF] and Caucasian (*n* = 9) adults 42.0 ± 9.8 years with hypertension (139.8 ± 10.4/84.6 ± 6.2 mmHg) performed three random experiments: bouts of vigorous and moderate intensity cycling and control. Subjects were attached to an ambulatory BP monitor for 19 h. We performed deep‐targeted exon sequencing with the Illumina TruSeq Custom Amplicon kit. Variant genotypes were coded as number of minor alleles (#MA) and selected for additional statistical analysis based upon Bonferonni or Benjamini–Yekutieli multiple testing‐corrected *P*‐values under time‐adjusted linear models for 19 hourly BP measurements for each subject. After vigorous intensity over 19 h, among *NOS3* variants passing multiple testing thresholds, as the #MA increased in rs891512 (*P* = 6.4E‐04), rs867225 (*P* = 6.5E‐04), rs743507 (*P* = 2.6E‐06), and rs41483644 (*P* = 2.4E‐04), systolic (SBP) decreased from 17.5 to 33.7 mmHg; and in rs891512 (*P* = 9.7E‐05), rs867225 (*P* = 2.6E‐05), rs41483644 (*P* = 1.6E‐03), rs3730009 (*P* = 2.6E‐04), and rs77325852 (*P* = 5.6E‐04), diastolic BP decreased from 11.1 mmHg to 20.3 mmHg among AF only. In contrast, after moderate intensity over 19 h in *NOS3* rs3918164, as the #MA increased, SBP increased by 16.6 mmHg (*P* = 2.4E‐04) among AF only. *NOS3* variants exhibited associations with PEH after vigorous, but not moderate intensity exercise among AF only. *NOS3* should be studied further for its effects on PEH in a large, ethnically diverse sample of adults with hypertension to confirm our findings.

## Introduction

Hypertension is the most common, costly, and preventable cardiovascular disease (CVD) risk factor, affecting 86 million (34%) adults in the United States and 1.4 billion (31%) adults in the world (World Health Organization 2013; Benjamin et al. 2017). By 2030, it is estimated that 41% of adults in the United States will have hypertension, and about an equal number will have prehypertension (Benjamin et al. 2017). Keeping this growing and costly public health crisis in check with the adoption and maintenance of lifestyle interventions such as habitual physical activity is a national and global priority (World Health Organization 2013, Egan et al. 2014). Aerobic exercise training lowers blood pressure (BP) from 5 to 7 mmHg among adults with hypertension (Pescatello et al. 2015a,b). Therefore, professional organizations throughout the world recommend regular participation in aerobic exercise for the prevention, treatment, and control of hypertension (Pescatello et al. 2015a).

Using ambulatory BP monitoring, we were the first to show that isolated exercise sessions (i.e., short‐term or acute) produce immediate but transient BP reductions of 5‐7 mmHg that persist for up to 24 h after the exercise session (Pescatello et al. 1991), a response termed *post‐exercise hypotension* (PEH) (Fitzgerald 1981; Kenney and Seals 1993). We and others posit that the chronic BP reductions resulting from aerobic exercise training are largely due to PEH (Fitzgerald 1981; Wilcox et al. 1982; Pescatello et al. 1991, 2004; Haskell 1994; Halliwill 2001; Pescatello and Kulikowich 2001; Thompson et al. 2001; Collier et al. 2008), although the mechanisms that mediate the acute and chronic exercise BP effects may differ (Pescatello et al. 2004; Green et al. 2014; Bruneau et al. 2016). PEH is of similar magnitude to the BP reductions that result from exercise training (Meredith et al. 1990; Jennings et al. 1991; Pescatello and Kulikowich 2001; Murray et al. 2006; Moker et al. 2014), and strongly correlates with the BP response to exercise training (Liu et al. 2012; Hecksteden et al. 2013; Tibana et al. 2015). For these reasons, PEH is a time‐efficient and clinically relevant model to investigate the antihypertensive effects of aerobic exercise.

Despite the documented antihypertensive benefits of acute (i.e., PEH) and chronic (i.e., training) aerobic exercise, there is significant interindividual variability in the BP response to exercise. Investigators from the *HE*alth, *RI*sk Factors Exercise *T*r*A*ining and *GE*netics or *HERITAGE* Family Study involving over 700 White and Black subjects have established the BP response to acute and chronic aerobic exercise is heritable (*h *=* *0.13‐0.42), with evidence of a shared genetic influence (Hagberg et al. 1999, 2000; Rankinen et al. 2000a,b; Rankinen et al. 2002, 2007; Bruneau et al. 2016; Rice et al. 2002; Bouchard and Rankinen 2001). Over the past 15 years, we (Augeri et al. 2009; Olson et al. 2012; Ash et al. 2013a; Bruneau et al. 2016; Pescatello et al. 2016) and others (Rankinen et al. 2000a,b, 2002; Rauramaa et al. 2002; Flavell et al. 2006; Hautala et al. 2006; Feairheller et al. 2009; Vargas et al. 2013) have been investigating candidate genes that account for the variability in the BP response to exercise.

Because of its documented role in the etiology of hypertension and regulation of exercise performance, we completed several studies investigating the influence of the endothelial nitric oxide synthase (*NOS3*) single‐nucleotide variant (SNV) ‐786 T>C (rs2070744) on PEH at varying exercise intensities among 50 Caucasian men with pre‐ to Stage 1 hypertension (Augeri et al. 2009; Olson et al. 2012; Ash et al. 2013a). In these studies, we found that PEH was of greater magnitude among men who were carriers of the *NOS3* C^786^ minor allele (MA) after light and moderate intensity than men with the *NOS3* TT genotype (Augeri et al. 2009; Ash et al. 2013a). Yet, carriers of the *NOS3* C^786^ MA had a more exaggerated peak systolic BP (SBP) response to a maximal graded exercise test (GEST) than men with the *NOS3* TT genotype (Olson et al. 2012). A significant limitation of this candidate gene approach is that initial findings of BP‐genotype associations often are not confirmed in follow‐up studies (Bouchard 2011; Bouchard et al. 2012; Ash et al. 2013a,b; Bruneau et al. 2016; Mattsson et al. 2016). Therefore, in a replication cohort, we utilize genomic technology that was not available when we performed our discovery phase candidate gene association studies to confirm if *NOS3* variants exhibit exercise intensity‐dependent associations with PEH.

## Materials and Methods

### Overview

**Figure 1:**
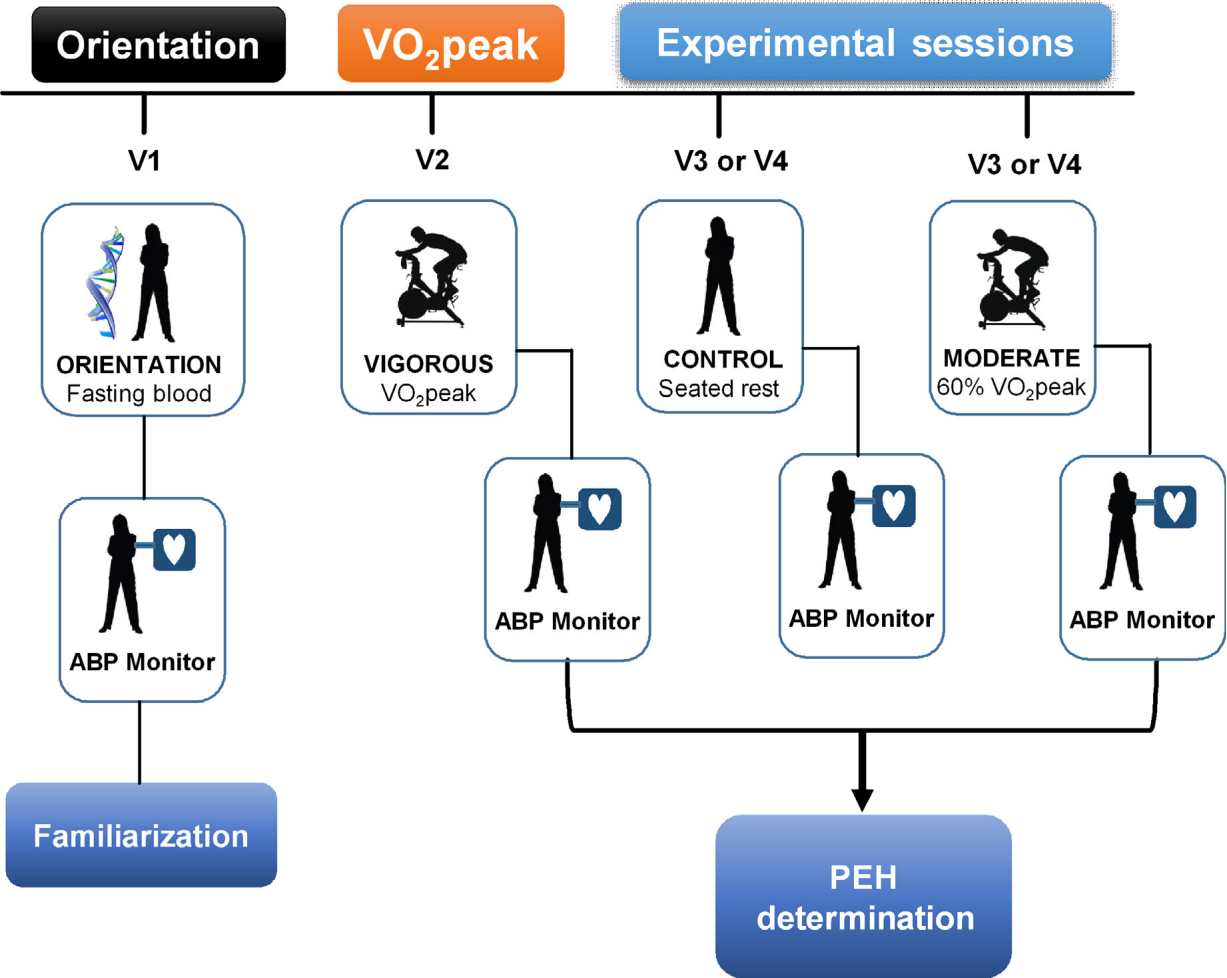
Study design overview. ABP, ambulatory blood pressure worn until the next morning; VO_2_peak, peak oxygen consumption as determined on the peak cardiopulmonary‐graded exercise stress test.

We employed the same study design as in our previous PEH studies that is overviewed in Figure 1 (Augeri et al. 2009; Olson et al. 2012; Ash et al. 2013a; Pescatello et al. 2016). At the orientation session, subjects (*n* = 23) provided a blood sample for deep‐targeted exon sequencing and a fasting cardiometabolic profile. They exited the laboratory wearing an ambulatory BP monitor until the next morning to acquaint them with the technology (Ash et al. 2013a,b). Subjects then completed three randomly assigned acute experiments: a cardiopulmonary graded exercise test (GEST) on a cycle ergometer to measure peak oxygen consumption (*V*O_2peak_) (VIGOROUS); 30 min of cycling at 60% *V*O_2peak_ (MODERATE); and a 30‐min control session of seated rest (CONTROL). We measured BP for 20 min before and 45 min after these experiments. After the experiments, subjects left the laboratory wearing an ambulatory BP monitor for 19 h until the next morning.

### Subjects

Subjects were 18 to 55 years old, sedentary (i.e., exercising ≤2 days per week), overweight to obese [i.e., body mass index (BMI) ≥25 to <40 kg•m^−2^], and had pre‐ to stage 1 hypertension. Subjects self‐reported their race/ethnicity as Caucasian or African American (AF). Any medications that could possibly influence BP were stopped at least 4 weeks prior to testing. These medications included inhaled or oral steroids, nonsteroidal anti‐inflammatory agents, aspirin, antihypertensive and hyperlipidemic medications, nutritional supplements besides a one‐a‐day vitamin, cold medications, hormone‐altering contraception, or herbal supplements. Subjects with osteoarthritis and orthopedic problems were not enrolled if these conditions limited their ability to complete the exercise experiments. With physician permission, two subjects discontinued their antihypertensive medications ≥6 weeks prior to study participation. Women were premenopausal and had regular menstrual cycles. Subject weight was monitored throughout study participation to ensure they were weight stable (i.e., gaining or losing <2.25 kg of orientation body weight). Participants completed an informed consent approved by the Institutional Review Boards of the University of Connecticut and Hartford Hospital.

### Body composition

BMI (kg•m^−2^) was determined from body weight and height with a calibrated balance beam scale. Waist circumference was taken with a nondistensible Guilick tape measure at the narrowest part of the torso (Pescatello et al. 2013).

### Blood pressure

At the orientation session, BP was measured with standard procedures (Pickering et al. 2005) using an automated BPTRU monitor (BPTRU Medical Devices; Coquitlam, Canada) to determine BP status. BP was also measured before the experiments every 2 min for 20 min in the nondominant arm with the automated BPTRU monitor and averaged as baseline BP. After the orientation session and the three experiments (i.e., CONTROL, MODERATE, VIGOROUS) using our previous protocols (Augeri et al. 2009; Olson et al. 2012; Ash et al. 2013a; Pescatello et al. 2016), subjects were attached to the same Oscar2 ambulatory BP monitor (Oscar2 automatic noninvasive ambulatory BP monitor, Suntech Medical Instruments Inc., Raleigh, NC) that was calibrated to a mercury sphygmomanometer. The ambulatory BP monitor was programmed to record three ambulatory BP assessments per waking hour and two per sleeping hour 19 h.

While wearing the monitor, subjects were instructed to proceed with their normal daily activities and not engage in formal exercise. They carried a journal, recording activities performed during the measurements, any unusual physical or emotional events, and their awake and sleep time. We excluded ambulatory BP readings of systolic BP (SBP) >220 or <80 mmHg, or diastolic BP (DBP) >130 or <40 mmHg according to the manufacturer's exclusion criteria. Ambulatory BP reports were acceptable if at least 80% of the potential BP readings were obtained. We calculated ambulatory arterial stiffness index (AASI) after CONTROL as 1 – (slope of DBP vs. SBP over 19 h) (Dolan et al. 2006).

### Acute experiments

Subjects performed three randomly assigned acute experiments: a nonexercise control session of seated rest (CONTROL) and two cycle exercise bouts on an upright cycle ergometer (Monarch 839E Digital Cycle Ergometer, Stockholm, Sweden) at 60% (MODERATE) and 100% (VIGOROUS) *V*O_2peak_ (Figure 1). The three experiments were conducted at the same time of day to account for the diurnal variation in BP, separated by a minimum of 48 h to preclude acute exercise effects, and finished within 1 month of starting the study. As in our PEH discovery phase studies, subjects were instructed to consume a standard breakfast 2 to 3 h before all experiments consisting of 250 mL orange juice, 125 mL skim or 1% fat milk, and either 125 mL of plain cereal such as cornflakes, two slices white toast, one English muffin, or one bagel 9 cm in diameter. They were also instructed to refrain from caffeinated beverages for 6 h before all experiments. Subjects lightly held the handle bars of the cycle ergometer during MODERATE and VIGOROUS. The same investigator measured heart rate (HR), SBP, and DBP for all subjects and experiments. Subjects sat quietly for a 20‐min baseline period at the beginning of each experiment. During the baseline period, HR was recorded with a HR monitor (Polar Electro Inc., Port Washington, NY) every 2 min, while SBP and DBP were obtained every other minute by auscultation. A 45‐min recovery period in the seated position followed each experiment with BP and HR obtained every 2 min. Subjects were attached to the ambulatory BP monitor after the experiments and removed it when they awoke the following morning.

VIGOROUS (100% *V*O_2peak_) was a peak cardiopulmonary GEST. *V*O_2peak_ was determined by breath‐by‐breath analysis of expired gases (ParvoMedicsTrueOne^®^ 2400 Metabolic Measurement System, ParvoMedics Inc., Sandy, UT). The GEST consisted of continuous cycling at a constant cadence (60 rev/min) beginning with a resistance of 0.5 kp (30 W) that was increased 0.5 kp every 2 min until volitional exhaustion. During the GEST, HR was recorded continuously with a 12‐lead electrocardiograph (Marquette Case 8000, Jupiter, FL), and BP was obtained every 2 min by auscultation. Results of the peak cardiopulmonary GEST (VIGOROUS) were used to calculate the workload of the other exercise experiment (MODERATE). Subjects performed the two remaining experiments in random order: nonexercise control and MODERATE (60% *V*O_2peak_). CONTROL consisted of 30 min of seated rest. MODERATE consisted of a 5‐min warm up of cycling with no resistance, 20 min of cycling at 60% *V*O_2peak_, and a 5‐min cool down to total 30 min. HR, SBP, and DBP were measured at 5‐min intervals during nonexercise control and MODERATE.

### Blood sampling and analysis

During the orientation session, fasting blood samples were drawn without stasis from an antecubital vein and centrifuged at 3400*g* at 23°C for 10–15 min. Serum was drawn in red top and plasma samples in EDTA vacutainer tubes. Serum and plasma samples were aliquoted into separate 1.8 mL nonpyrogenic storage tubes and frozen at −80°C for future analysis. Glucose and insulin were determined by enzymatic/spectrophotometric methods from which the homeostasis model assessment, an insulin resistance biomarker, was calculated (Matthews et al. 1985). Total cholesterol, triglycerides, and high‐density lipoprotein cholesterol were determined by enzymatic/spectrophotometric methods, and low‐ density lipoprotein cholesterol was calculated with the Friedewald equation (Friedewald et al. 1972). Nitrite (NO_2‐_)/Nitrate (NO_3‐_), high‐sensitivity C‐reactive protein (CRP), endothelin 1‐21, and plasma renin activity (PRA) were measured by enzymatic/spectrophotometric methods. Blood analyses were performed with two levels of quality control. A blood sample for DNA was drawn in an EDTA purple top vacutainer tube that was centrifuged for white cell isolation and frozen at −80°C for future DNA extraction.

### Targeted sequencing and variant calling

We then performed deep‐targeted exon sequencing of a prioritized panel of 41 genes that contained polymorphisms *reported* to be associated with hypertension, the BP response to pharmacotherapy, and/or the BP response to PEH and exercise training (Ash et al. 2013a; Bruneau et al. 2016) using the Illumina TruSeq Custom Amplicon kit (Catalog# FC‐130‐1001, Illumina, San Diego, CA) (Pescatello et al. 2016). The Illumina DesignStudio software was used to create probes for the generation of 1214 amplicons with a size range of 225–275 bp. The TruSeq Custom Amplicon manifest file associated with this prioritized panel of genes included Target ID, region, chromosome, and start and end hg19 reference coordinate positions. Following the TruSeq Custom Amplicon Library Preparation Guide, sequencing libraries were prepared. For all libraries, DNA input mass was 250 ng of DNA. Libraries were generated with dual indices (23 PCR cycles) followed by normalization and pooling. The library amplicon pool was sequenced with Illumina MiSeq version 2 reagents (250 paired‐end reads). From the library amplicon pool, 7.1 million pair‐end reads (6.8 million passing quality filter) were produced. MiSeq Reporter Software (version 2.3.32), using the TruSeq Amplicon workflow, generated Fastq files and aligned reads to the hg19 human reference sequence with the Smith‐Waterman algorithm. The Genome Analysis Toolkit (GATK) was used for variant calling (SNVs and small insertion/deletions) and the creation of variant calling files (VCF). For all further downstream analysis, a merged VCF was generated with VCFtools v 0.1.12b (Danecek et al. 2011) and custom R scripts (R v3.2.0). Only variants with FILTER=PASS were retained. For each defined amplicon target region, we calculated the total number of variants present per subject and each polymorphism's major allele and MA frequency for each subject.

### Statistical analysis

Descriptive statistics (Mean±SD) were obtained on study variables for the total sample and by ethnic group. Independent t‐tests determined if there were differences in subject descriptive characteristics between AF and Caucasians. Repeated measures analysis of covariance tested if the BP response, defined as the change from baseline following exercise – change from baseline following control at hourly intervals under ambulatory conditions, differed over 19 h with age and BMI as covariates and gender and ethnicity as fixed factors. These statistical analyses were performed with SPSS 14.0 (Chicago, IL).

#### Variant Screening

Variant genotypic values were coded as the number MA (#MA). Genotypic values for 645 variants from the 41 genes were analyzed (Pescatello et al. 2016). For each polymorphism, we fit a linear model for each race/ethnicity separately that included polynomial time (order 3), polymorphism under an additive model, and polymorphism x time interactions as covariates, and the dependent variable, BP response. Since there are 19 observations per subject (i.e., *n* = 9 Caucasians × 19 h = 171 observations; *n* = 14 AF × 19 h = 266 observations), we assumed a first‐order autoregressive (AR1) correlation structure. Residual errors within each subject are thus assumed correlated, but assumed independent across subjects. Bonferroni and Benjamini–Yekutieli (BY) (Benjamini and Yekutieli 2005) adjusted *P*‐values were calculated for each polymorphism, correcting for the total number of unique polymorphism profiles among each racial/ethnic group resulting in 300 polymorphisms for AF and 146 for Caucasians. Polymorphisms with Bonferroni‐adjusted *P*‐values < 0.05 and/or BY‐adjusted *P*‐values < 0.25 were identified; *NOS3* polymorphisms achieving these multiple testing thresholds were then considered for further statistical modeling and analysis. *NOS3* genotype‐BP differences by the #MA after VIGOROUS and MODERATE compared to CONTROL are reported as the average change over 19 h with the associated p value resulting from the screening model that accounted for repeated measures over time.

#### Final multivariable regression models

For each *NOS3* polymorphism achieving the multiple testing threshold, we selected model effects (i.e., covariates, order of time polynomial, additive vs. dominant/recessive genetic models) and within‐subject correlation structure based on models fit with maximum likelihood (ML) using Akaike Information Criteria and likelihood ratio tests (LRT). Covariates that were marginally associated (*P* < 0.05) with the BP response were eligible to be included in the final models. Possible within‐subject correlation structures included compound symmetry, AR1, and independent structures; however, AR1 provided the best fit in all cases. Based on ML estimation using AR1 within‐subject correlation structure, we report the LRT *P*‐values and pseudo‐partial R‐squared measures (i.e., the partial proportion of variation explained [PVE]) for the polymorphism effects.

We defined the PVE for a given model using the pseudo‐R‐squared measure R^2 ^_m_= 1‐(L_R_/L_U_)^2/n^, where L_R_ is the restricted maximized likelihood from a model containing only an intercept, L_U_ is the unrestricted maximized likelihood for the given model, and n is the number of subjects (Maddala 1983; Magee 1990). The partial PVE for each polymorphism was the difference between R^2^_m_ for the final model (including the covariate and polymorphism effects) and R^2^_m_ for a model with polymorphism effect(s) excluded (Schemper 1993). We also report the parameter estimates for the final models, obtained using the restricted maximum likelihood (REML) method. Statistical analysis was performed in R (screening) and SAS version 9.4 (final models).

### Annotation assessment of causal variation and regulatory effects

For each polymorphism that passed the multiple testing threshold, we determined the combined annotation‐dependent depletion (CADD) score from www.cadd.gs.washington.edu (Kircher et al. 2014). CADD scores quantitatively prioritize functional, deleterious, and disease causal variants across a wide range of functional categories, effect sizes, and genetic architectures. The higher the CADD score, the more severe the allelic substitution in terms of its causal variation. CADD scores of ≥10 indicate that substitutions in a polymorphism are predicted to be the 10% most deleterious substitutions in the human genome. We also searched ENCODE‐based datasets to infer regulatory effects for each polymorphism passing multiple testing thresholds using the Chromatin Immunoprecipitation Coupled to Massively Parallel Sequencing (ChIP‐seq) annotations across ENCODE cell lines and the human reference lymphoblast cell line GM12878 hg19. Genome segmentation data for hg19 is based on 25 different chromatin states (including eight different chromatin marks, RNA polymerase II, and CTCF binding) that are used to segment the genome using both ChromHMM and Segway (ENCODE Project Consortium 2012, Hoffman et al. 2013).

## Results

### Subjects

Subject characteristics are displayed in Table 1 (Pescatello et al. 2016). Subjects (*n* = 23) were middle‐aged, obese Caucasian (39%) and AF (61%) men (*n* = 10) and women (*n* = 4) with pre‐ to established hypertension. More AF (64.3%) reported a family history of hypertension than did the Caucasians (22%) (*P* = 0.049). The cardiometabolic health profile of the AF was more favorable than the Caucasians; however, only waist circumference (*P* = 0.015), total cholesterol (*P* = 0.027), and triglycerides (*P* = 0.003) were different between ethnic groups. When compared to reference standards, NO_2‐_/NO_3‐_ (Ghasemi et al. 2010) were low among AF and normal–high among Caucasians; CRP was high among AF and Caucasians (http://lsplinks.net/QUESTCRP); and endothelin‐1‐21 (http://lsplinks.net/ALPCOBig) and PRA (Brossaud and Corcuff 2009) were normal among AF and Caucasians.

**Table 1:** Subject Characteristics (X±SD) (Pescatello et al. 2016)

Variable	Caucasians (*n* = 9)	African American (*n* = 14)
Age (yr)	45.1 ± 7.8	39.9 ± 10.6
Body mass index (kg·m^−2^)	30.5 ± 1.8	31.1 ± 4.5
Waist circumference (cm)	98.0 ± 7.2	88.3 ± 9.4*
Relative peak oxygen consumption (mL·kg^−1^·min^−1^)	29.7 ± 6.4	25.3 ± 5.7
Awake systolic blood pressure (mmHg)	139.3 ± 7.0	140.2 ± 12.3
Awake diastolic blood pressure (mmHg)	85.0 ± 5.1	84.3 ± 6.9
Glucose (mg∙dL^−1^)	96.4 ± 12.2	97.2 ± 10.3
Ambulatory arterial stiffness index	0.415 + 0.93	0.391 + 0.143
Insulin (ulU∙mL^−1^)	13.1 ± 10.1	9.4 ± 6.0
Homeostatic Model of Assessment	3.1 ± 2.2	2.3 ± 1.5
Total cholesterol (mg∙dL^−1^)	207.8 ± 31.3	178.5 ± 27.3*
Low‐density lipoproteins (mg∙dL^−1^)	129.4 ± 20.3	108.3 ± 32.7
High‐density lipoproteins (mg∙dL^−1^)	44.1 ± 10.9	53.9 ± 14.8
Triglycerides (mg∙dL^−1^)	170.8 ± 88.8	83.7 ± 35.8**
Nitrite (NO_2_^−^)/Nitrate (NO_3_^−^) (*μ*mol∙L^−1^)	23.3 ± 37.0	10.9 ± 13.1
C‐reactive protein (mg∙dL^−1^)	1.1 ± 1.0	2.8 ± 3.5
Endothelin (pmol∙L^−1^)	0.222 ± 0.213	0.378 ± 0.663
Plasma renin activity (ng∙mL^−1^∙hr^−1^)	1.7 ± 1.09 (*n* = 2)	0.946 ± 0.840 (*n* = 8)

*P < 0.05; **P < 0.01.

### Blood pressure response

Overall, among the total sample, the SBP and DBP responses over 19 h were not different after VIGOROUS (SBP/DBP, −0.7 ± 13.4/−0.6 ± 7.7 mmHg) or MODERATE (−3.1 ± 8.0 mmHg/−2.5 ± 5.7) compared to control (*P* > 0.05) (Pescatello et al. 2016). Furthermore, the SBP and DBP responses over 19 h were not different between Caucasians versus AF after VIGOROUS (SBP/DBP, 0.6 ± 10.1/−0.2 ± 5.3 mmHg vs. −1.5 ± 15.5/−0.9 ± 9.1 mmHg) or MODERATE (−3.0 ± 6.6/−1.6 ± 5.2 mmHg vs. −3.2 ± 9.0/−3.0 ± 6.1 mmHg), respectively, (*P* > 0.05).

#### By Number of *NOS3* Variant Minor Alleles

In contrast to the overall BP response, the SBP and DBP responses over 19 h after VIGOROUS and MODERATE compared to control by *NOS3* variant #MA passing multiple testing thresholds differed between Caucasians and AF. After VIGOROUS, as the #MA increased from 0 to 1 or 2 depending on the variant, SBP and/or DBP decreased after VIGOROUS versus control over 19 h (Table 2). For, *NOS3* rs891512, SBP decreased by −30.4 mmHg (*P* = 6.4E‐04) and DBP decreased by −16.3 mmHg (*P* = 9.7E‐05); rs867225, SBP decreased by −17.5 mmHg (*P* = 6.5E‐04) and DBP decreased by −11.7 mmHg (*P* = 2.7E‐05); rs743507, SBP decreased by −21.3 mmHg (*P* = 2.6E‐06); rs41483644, SBP decreased by −33.7 mmHg (*P* = 2.4E‐04) and DBP decreased by −17.6 mmHg (*P* = 1.6E‐03); rs3730009, DBP decreased by −11.9 mmHg (*P* = 2.6E‐04); and rs77325852, DBP decreased by −11.1 mmHg (*P* = 5.6E‐04) among AF but not Caucasians. In contrast, after MODERATE, as the #MA for rs3918164 increased from 0 (*n* = 12, −5.6 ± 6.9 mmHg) to 1 (*n* = 2, 10.9 ± 7.3 mmHg), SBP increased +16.6 mmHg (*P* = 2.4E‐0.4) after exercise versus control over 19 h among AF but not Caucasians. There were no other significant *NOS3* variant associations with the BP response passing multiple testing thresholds following MODERATE among AF or Caucasians.

**Table 2:** The blood pressure response (X±SD)^1^ after versus before **VIGOROUS** exercise compared to control over 19 h by Nitric Oxide Synthase 3 (*NOS3*) # of Minor Alleles Located on Chromosome (Chr) 7q36.1: 151,010,605‐151,011,105: 501 bp

			Racial/Ethnic Group					
			African American (*n* = 14)			Caucasians (*n* = 9)		
Variant	Regulatory Effects^2^	CADD Score	# Minor Alleles	SBP (mmHg)	DBP (mmHg)	# Minor Alleles	SBP	DBP
**rs891512**	Transcript Variant in an Intron Overlaps 19 transcripts and 2 regulatory features	4.468	0 (*n* = 13) 1 (*n* = 1) 2 (*n* = 0)	**0.6 ± 13.7** **−29.8 ± NA** NA	**0.6 ± 7.6** **−19.7 ± NA** NA	0 (*n* = 3) 1 (*n* = 5) 2 (*n* = 1)	3.0 **±** 7.6 −2.7 **±** 11.7 9.6 **±** NA	−1.6 **±** 3.5 0.9 **±** 6.9 −1.6 **±** NA
**rs867225**	Alternate Transcript Variant in an Intron Same Enhancer/Promoter Block as rs3730009 (K562 cells) Predicted Repressed/Low Activity in GM 12878	7.457	0 (*n* = 9) 1 (*n* = 5) 2 (*n* = 0)	**4.72 ± 9.5** **−12.8 ± 18.7** NA	**3.3 ± 5.4** **−8.4 ± 10.0** NA	0 (*n* = 9) 1 (*n* = 0) 2 (*n* = 0)	0.6 **±** 10.1 NA NA	−0.2 **±** 5.3 NA NA
**rs743507**	Transcript Variant in an Intron Overlaps 19 transcripts and 2 regulatory features	NA	0 (*n* = 9) 1 (*n* = 5) 2 (*n* = 0)	**6.1 ± 8.8** **−15.2 ± 16.1** NA	0.3 **±** 4.8 −2.9 **±** 14.6 NA	0 (*n* = 3) 1 (*n* = 5) 2 (*n* = 1)	3.0 **±** 7.6 −2.7 **±** 11.7 9.6 **±** NA	−1.6 **±** 3.5 0.9 **±** 6.9 −1.6 **±** NA
**rs41483644**	Transcript Variant in an Intron Noncoding Transcript Variant 3′ UTR Variant Overlaps 19 transcripts and 2 regulatory features	2.83	0 (*n* = 13) 1 (*n* = 1) 2 (*n* = 0)	**0.9 ± 13.1** **−32.8 ± NA** NA	**0.4 ± 8.1** **−17.2 ± NA** NA	0 (*n* = 9) 1 (*n* = 0) 2 (*n* = 0)	0.6 **±** 10.1 NA NA	−0.2 **±** 5.3 NA NA
**rs3730009**	Transcript Variant in an Intron Enhancer/Promoter (K562 cells) Predicted Transcribed in GM 12878	10.81	0 (*n* = 7) 1 (*n* = 6) 2 (*n* = 1)	5.9 **±** 10.5 −10.3 **±** 17.8 −1.2 **±** NA	**4.4 ± 5.6** **−5.9 ± 9.9** **−7.5 ± NA**	0 (*n* = 9) 1 (*n* = 0) 2 (*n* = 0)	0.6 **±** 10.1 NA NA	−0.2 **±** 5.3 NA NA
**rs77325852**	Alternate Transcript Variant in an Intron 3′ Downstream Variant Antisense Transcript for Autophagy Related 9 Homolog B (*ATG9B*) Involved with Posttranscriptional Regulation of *NOS3* Predicted Promotor Region in H1‐ESC, enhancer in GM 12878 and transcribed in K562	7.62	0 (*n* = 8) 1 (*n* = 5) 2 (*n* = 1)	5.6 **±** 9.8 −13.0 **±** 18.6 −1.2 **±** NA	**3.6 ± 5.7** **−6.7 ± 10.9** **−7.5 ± NA**	0 (*n* = 9) 1 (*n* = 0) 2 (*n* = 0)	0.6 **±** 10.1 NA NA	−0.2 **±** 5.3 NA NA

SBP, Systolic Blood Pressure; DBP, Diastolic Blood Pressure; UTR, Untranslated Region; NA, Not Available.

^1^X and SD are computed as the average and standard deviation, respectively, of the subject‐level BP response averaged over 19 h. Bolded values correspond to significant effects after multiple testing adjustments.

^2^Chromosome location and regulatory effects obtained from ENCODE‐based datasets with Chromatin Immunoprecipitation Coupled to Massively Parallel Sequencing (ChIP‐seq) annotations across six human cell lines to segment the genome (hg19) based on 25 different states, merging ChromHMM and Segway analyses.

SNAP = SNP Annotation and Proxy Search www.broadinstitute.org/mpg/snap/ldsearch.php;

University of California Santa Cruz UCSC Genome Brower http://www.genome.ucsc.edu/; and Ensembl http://grch37.ensembl.org/index.html

CADD=Combined Annotation Dependent Depletion, a score that prioritizes causal variation and regulatory effects http://cadd.gs.washington.edu/score

### Proportion of variance explained

Table 3 contains the PVE for the SBP and DBP response to VIGOROUS and MODERATE among AF for the final multivariable regression models without the *NOS3* variants, and the partial PVE for each *NOS3* polymorphism after accounting for the other covariates in the model. For the SBP response to VIGOROUS, resting ambulatory SBP over 19 h, age, resting AASI over 19 h, and time (order 3) accounted for 92.5% of the variation. When the other covariates in the model were accounted for, the individual *NOS3* variants explained up to 5.6% (*P* = 0.0006) of the variation. For the DBP response to VIGOROUS, fasting triglycerides, gender, and endothelin accounted for 85.8% of the variation. When the other covariates in the model were accounted for, the individual *NOS3* variants explained up to 7.6% (*P* = 0.0011) of the variation. For the SBP response to MODERATE, insulin accounted for 66.2% of variation. When insulin was accounted for, *NOS3* rs3918164 explained an additional 4.1% (*P* = 0.1797) of the variation.

**Table 3:** The proportion of variance explained in the multivariable regression models for the systolic and diastolic blood pressure response following VIGOROUS and MODERATE among African American, with and without *NOS3* polymorphisms passing multiple testing thresholds

		**VIGOROUS**		
**SBP**				
	**Polymorphism**	**Model** 1	**PVE** 2	***P*** **‐value** 3
	None	BP Response = −1.7872 + 2.1590*time + 0.0207*time^2 ‐ 0.0320*time^3 + 16.4343*log(AASI) + 0.6782*Orientation 19 h SBP + 0.5765*Age	0.9252	–
	**Polymorphism**	**Model** 1	**Partial PVE** 2	***P*** **‐value** 3
	rs891512	BP Response = −0.2841 + 1.5349*time + 0.0091*time^2 ‐ 0.0210*time^3 + 11.1560*log(AASI) + 0.6298*Orientation 19 h SBP + 0.5368*Age ‐ 18.5855*SNP + 8.5552*SNP*time + 0.0618*SNP*time^2 ‐ 0.1505*SNP*time^3	0.0562	0.0006
	rs867225	BP Response = 1.4194 + 2.1533*time + 0.0175*time^2 ‐ 0.0319*time^3 + 13.9592*log(AASI) + 0.6356*Orientation 19 h SBP + 0.4446*Age – 8.7596*SNP	0.0225	0.0253
	rs743507	BP Response = 1.1083 + 2.1574*time + 0.0198*time^2 ‐ 0.0319*time^3 + 10.0134*log(AASI) + 0.5465*Orientation 19 h SBP + 0.4805*Age – 8.0473*SNP	0.0109	0.1380
	rs41483644	BP Response = −0.1207 + 2.1486*time + 0.0149*time^2 ‐ 0.0318*time^3 + 15.8916*log(AASI) + 0.6262*Orientation 19 h SBP + 0.3839*Age ‐ 21.3422*SNP	0.0358	0.0026
**DBP**				
	**Polymorphism**	**Model** 1	**PVE** 2	***P*** **value** 3
	None	BP Response = −3.1187 ‐ 4.3889*log(Endothelin) ‐ 8.5055*log(TRIG) + 7.6938*Gender	0.8577	–
	**Polymorphism**	**Model** [Link]	**Partial PVE** 2	***P*** **value** 3
	rs891512	BP Response = −1.7776 ‐ 4.0777*log(Endothelin) ‐ 5.5915*log(TRIG) + 6.8446*Gender ‐ 15.2493*SNP	0.0760	0.0011
	rs867225	BP Response = −1.1661 – 3.3220*log(Endothelin) – 7.7889*log(TRIG) + 6.4164*Gender – 4.4431*SNP	0.0172	0.1797
	rs41483644	BP Response = −2.9066 ‐ 4.1740*log(Endothelin) – 8.5088*log(TRIG) + 7.3595*Gender – 1.6472*SNP	0.0010	0.7518
	rs3730009	BP Response = −1.0641 – 3.9416*log(Endothelin) – 6.4003*log(TRIG) + 6.6305*Gender – 3.0620*SNP	0.0172	0.1797
	rs3730009^4^	BP Response = −0.4655 – 3.7484*log(Endothelin) ‐7.1585*log(TRIG) + 6.0830*Gender – 4.3820*SNPr	0.0241	0.1068
	rs77325852	BP Response = −1.0567 – 3.8448*log(Endothelin) – 7.4939*log(TRIG) + 6.4025*Gender – 3.3822*SNP	0.0250	0.1003
	rs77325852^4^	BP Response = −0.0329 – 3.4764*log(Endothelin) – 8.9648*log(TRIG) + 5.4137*Gender – 5.6710*SNPr	0.0391	0.0339
		**MODERATE**		
**SBP**				
	**Polymorphism**	**Model** 1	**PVE** 2	***P*** **value** 3
	None	BP Response = −3.2665 + 10.9273*log(INSULIN)	0.6623	–
	**Polymorphism**	**Model** 1	**Partial PVE** 2	***P*** **value** 3
	rs3918164	BP Response = −4.4056 + 7.4821*log(INSULIN) + 7.9659*SNP	0.0407	0.1797

NOS3, Endothelial Nitric Oxide Synthase; VIGOROUS, 100% of peak oxygen consumption (*V*O_2peak_); MODERATE, 60% *V*O_2peak_; SBP, systolic blood pressure; DBP, diastolic blood pressure; PVE, proportion of variance explained; SNP, polymorphism; TRIG, triglycerides; AASI, resting ambulatory arterial stiffness index over 19 h; Orientation 19 h SBP = resting ambulatory SBP over 19 h.

^1^Restricted maximum likelihood estimates reported; all covariates centered except for polymorphism.

^2^Either polymorphism only (when there is no polymorphism by time interaction) or joint polymorphism and polymorphism by time effects (when there is a polymorphism by time interaction), computed using maximum likelihood.

^3^Raw (unadjusted) p values from likelihood ratio tests for either polymorphism only (when there is no polymorphism by time interaction) or joint polymorphism and polymorphism by time effects (when there is a polymorphism by time interaction) under maximum likelihood.

^4^Dominant model for polymorphism (i.e., SNPr=0 if 0 copies of the minor allele; SNPr=1 if 1 or 2 copies of the minor allele; additive genetic models used for all other polymorphisms (i.e., SNP= #minor allele).

### Annotation assessment of causal variation and regulatory effects

The CADD scores for variants passing the threshold for multiple testing for VIGOROUS are displayed in Table 2. *NOS3* rs3730009 had a CADD score of 10.81, rs77325852 had a CADD score of 7.62, and rs867225 had a CADD score of 7.457. In addition, for MODERATE, rs3918164 had CADD score of 7.26. Therefore, these transcript variants in an intron are likely to have causal variation effects (Kircher et al. 2014). Using the ENCODE‐based datasets, the ChIP‐seq annotations for VIGOROUS shown in Table 2 provide strong evidence of regulatory effects for several of the variants based on the segmentation analyses across the human genome. For example, rs773225852 is an antisense transcript for autophagy‐related 9 homolog B (*ATG9B*) that is involved in posttranscriptional regulation of *NOS3* and is in a strong promoter region; and rs867225 is a region of predicted repressed/low activity in GM 12878, but is an alternate transcript in the same enhancer/promoter block as rs3730009. For MODERATE, rs391816 (not shown in Table 2) is in a low‐activity region across many cancer lines, predicted repressed/low activity in GM 12878, and is a proxy for Potassium Voltage‐Gated Channel Subfamily H Member 2 (*KCNH2*, OMIM 152427).

## Discussion

Because of published reports that *NOS3* rs1799983 (+894 G>T, OMIM +163729), rs2070744 (‐786 T>C), and rs1800779 (‐922 A>G) were associated with hypertension and the BP response to antihypertensive pharmacotherapy and/or exercise training, over a decade ago, we began several discovery phase candidate gene association studies for their associations with PEH (Augeri et al. 2009; Olson et al. 2012; Ash et al. 2013a). In these earlier studies, we found that *NOS3* rs2070744 (‐786 T>C) had exercise intensity‐dependent associations with PEH among overweight to obese, Caucasian men with pre‐ to established hypertension. The major findings from this validation study were that *NOS3* rs891512, rs867225, rs743507, rs41483644, rs3730009, and rs77325852 associated with PEH after VIGOROUS but not MODERATE among AF only. Among these *NOS3* variants passing multiple testing thresholds, after VIGOROUS over 19 h, as the #MA increased, SBP *decreased* by 18–34 mmHg and DBP *decreased* 11–20 mmHg after VIGOROUS compared to control. The magnitude of these BP reductions rival that of antihypertensive pharmacotherapy (Chobanian et al. 2003; James et al. 2014), can reduce the risk of CVD by 20% to 40% (Whelton et al. 2002a; Chobanian et al. 2003), and are similar in magnitude to those we recently reported for variants in the following renal genes: angiotensinogen‐converting enzyme (*ACE*), angiotensin type 1 receptor (*AGTR1*), aldosterone synthase (*CYP11B2*), and adducin (*ADD1*) (Pescatello et al. 2016). In contrast, in *NOS3* rs3918164 after MODERATE over 19 h, as the #MA increased, SBP *increased* by 17 mmHg among AF only. These *NOS3* variants passing multiple testing thresholds explained up to 8% of the variance in the BP response to acute aerobic exercise, a partial PVE that is larger than that typically reported for individual variants in exercise genomic studies examining health‐related phenotypes (Ash et al. 2013a; Bruneau et al. 2016). There were no significant *NOS3* variant associations with the BP response following VIGOROUS or MODERATE among Caucasians.

AF have the highest prevalence of hypertension of all ethnic groups at 46% (Benjamin et al. 2017; Rayner and Spence 2017). The progression to incident hypertension is more rapid among AF than Caucasians (Diaz et al. 2017; Egan 2017). AF are less likely to have their hypertension controlled, yet they use more antihypertensive medications than Caucasians (Whelton et al. 2002b; Benjamin et al. 2017; Carnethon et al. 2017). A recent meta‐analysis by Liu and colleagues (Liu et al. 2017) found that meeting the recommended minimum amount of physical activity of 150 min per week of moderate intensity or 75 min of vigorous intensity leisure‐time physical activity, reduced incident hypertension among adults with normal blood pressure who were largely Caucasian by 6%, with greater benefits accruing with increasing amounts of leisure‐time physical activity. The protective effects of physical activity on incident hypertension appear to be even greater for AF. Diaz et al. (2017) found that among AF who met or exceeded the recommended amount of physical activity, incident hypertension was reduced by 24%, substantiating the importance of regular participation in rigorous exercise among AF for cardiovascular health. Our new findings support observations from our earlier studies that *NOS3* variant MA carriers appear to experience the greatest BP reductions from exercise participation (Augeri et al. 2009). They are also consistent with the findings of Diaz et al. (2017) regarding the favorable effects of higher levels of physical exertion on BP among AF. Furthermore, the magnitude of PEH we observed would be sufficient to reduce the resting BP levels of the AF participants with pre‐ to established hypertension into normotensive ranges for the remainder of the day when BP is typically at its highest levels.

We and others have found that PEH differs between AF and Caucasians for reasons that are not clear (Headley et al. 1998; Pescatello et al. 2003; Santa‐Clara et al. 2003; Bond et al. 2005; Brandon and Elliott‐Lloyd 2006; Jones et al. 2006, 2007; Enweze et al. 2007; Yan et al. 2016). At rest, AF have increased carotid intima‐media thickness, lower vascular responsiveness to nitric oxide (NO), lower forearm blood flow, and higher forearm vascular resistance compared to Caucasians (Hinderliter et al. 1996; Heffernan et al. 2009; Ranadive et al. 2016). The key stimulus for the remarkable increases in muscle blood flow that occur with intense exercise is increased sheer stress within the vasculature that markedly increases *NOS3* expression and NO production and bioactivity. The potent vasodilatory actions of NO are implicated in the antihypertensive effects of aerobic exercise (Vimaleswaran et al. 2008; Augeri et al. 2009; Green et al. 2014). There is general agreement that aerobic exercise promotes arterial health in Caucasians; however, such information is lacking and conflicting in AF (Ranadive et al. 2016).

It is interesting to note that the partial PVE explained by the resting AASI in our final multivariable regression model for the SBP response to VIGOROUS, without *NOS3* variants in the model, was 6.3%, and the partial PVE explained by resting endothelin‐1‐21 levels, a potent vasoconstrictor, to the DBP response to VIGOROUS in the final multivariable regression model, without the *NOS3* variants in the model, was 43.5% (Table 3). Possible reasons for the ethnic differences in PEH by *NOS3* #MA that we observed may be partially attributed to interactions among the vasoactive properties of the endothelial nitric oxide synthase pathway as modulated by *NOS3* variant regulatory actions and exercise intensity. For, VIGOROUS may override the more vasoconstrictive nature of the vasculature of AF at rest in people who are in the early stages of hypertension such as our sample was, by increasing shear stress, *NOS3* expression, and NO production and availability, while simultaneously decreasing the production and availability of endothelin‐1‐21 and other vasoconstrictors; thereby, leading to immediate, clinically meaningful reductions in BP that persist for the remainder of the day (Augeri et al. 2009; Olson et al. 2012; Ash et al. 2013a). In support of this premise are our final multivariable regression models that showed resting AASI and endothelin‐1‐21 levels were positively correlated with the magnitude of PEH, suggesting that VIGOROUS overrode the greater vasoconstrictive nature of the vasculature of AF at rest via increased *NOS3* expression and NO production and availability, as well as by other peripheral and central mechanisms we did not measure.

Our new *NOS3* findings are consistent with our previous reports of exercise intensity‐dependent associations with *NOS3* rs2070744 (Augeri et al. 2009; Olson et al. 2012; Ash et al. 2013a) and renal variants in *ACE*,* AGTR1*,* CYP11B2*, and *ADD1* with PEH (Pescatello et al. 2016). Yet, although the same genes emerged from our discovery phase and validation studies regarding their associations with PEH (i.e., *NOS3*,* ACE*,* AGTR1*,* CYP11B2*, and *ADD1*), the individual variants located within them differed among our studies. It is also important to note that we were unable to confirm our original findings in Caucasians as we only observed significance among AF in this investigation. Reasons for these differences are unclear. Nonetheless, it appears that *NOS3* should be explored further for its regulatory effects on PEH.

A major limitation of this investigation is the small sample size, and so our findings should be taken with caution. For this reason, we calculated the power of the variant screening method using simulation based on the observed allele frequencies and estimated parameters from the screening model for each significant *NOS3* variant in Table 2 for VIGOROUS and *NOS3* rs3938364 for MODERATE. We then calculated the power to detect BP‐genotype significant differences as the proportion of 1000 simulations in which the variant *P*‐value (calculated using the residual degree of freedom method) was less than alpha=0.05/300 (i.e., adjusted for 300 unique genotypic profiles for AF). The power to detect the BP‐genotype significant differences ranged from 29.6% for rs41483644 (DBP) to 79.2% for rs743507 (SBP) in Table 2. In instances where there was insufficient power, for example, rs77325852 with a power of 30.5% for DBP and CADD score of 7.62 in Table 2, the CADD score indicated that this variant was likely to have causal variation effects. Evidence is mounting that noncoding variants located in DNA regulatory elements, as are all the *NOS3* variants that passed multiple testing thresholds in our analyses (Table 2), may have functional consequences by creating, deleting, or altering binding sites for transcriptional regulators (Lowdon and Wang 2017). Indeed, Chip‐seq annotations of *NOS3* rs373009, rs867225, and rs77325852 from ENCODE‐based datasets provide strong evidence supporting regulatory effects, however, linking a specific regulatory effect to these polymorphisms is beyond the scope of our investigation.

Another limitation of this study is the effect of exogenous sources of NO_2‐_/NO_3‐_ in foods (e.g., ham, vegetables, and roots) that can influence *NOS3* expression and NO production and availability. In order to minimize the confounding effects of diet on our findings, subjects were asked to maintain their normal diet throughout study participation as verified by weekly weigh‐ins, the only nutritional supplement that was allowed was a one‐a‐day vitamin, and subjects consumed a standard meal 2 to 3 h before all experiments. The purpose of instituting these procedures was to minimize the confounding effects of NO_2‐_/NO_3‐_ food sources on *NOS3* expression, and subsequently our findings.

In addition, we employed several methodological strategies to improve the statistical power to find *NOS3* genotype‐BP associations should they exist (Bouchard 2011; Ash et al. 2013a; Bruneau et al. 2016; Mattsson et al. 2016; Pescatello et al. 2016). Our strategies were use of: a repeated measure design that modeled the within‐subject correlation of the 19 hourly time points, a targeted inquiry of polymorphisms with a prioritized panel of genes that reduced the genomic search space, high‐throughput exon sequencing to target functional regions of the gene, and the same standardized protocols and methods in our discovery phase and replication studies. Other strengths of this replication study were inclusion of a randomized control design with the subjects serving as their own control; the clinic gold standard of BP assessment, ambulatory BP monitoring (Niiranen et al. 2014); and a well‐controlled, structured, and monitored exercise exposure; as well as adjustment for multiple testing that was based only on genetic variants exhibiting variability in the #MA and with unique genotypic values. Due to the small sample size, and particularly in instances when only one or two subjects carried a MA, the multivariable models adjusted for covariates may suffer from over fitting; thus, our findings should be interpreted with caution and regarded as preliminary.

In conclusion, in a replication cohort using high‐throughput exon sequencing, we found that *NOS3* variants once again showed exercise intensity‐dependent associations with PEH. This time, however, significance was found only among AF but not Caucasians as we originally reported. Therefore, it appears that *NOS3* genotype variation determines PEH following VIGOROUS but not MODERATE among AF. Our findings are limited by a small sample size so that they should be taken with caution. Yet, they add new and clinically important information to the exercise genomic literature regarding the immediate antihypertensive effects of exercise among AF, an ethnic group for which this information is significantly lacking. For this reason, the National, Heart, Lung, and Blood Institute has called for proof of concept studies such as ours conducted in other fields where treatment effects have been reported to differ by race (Whelton et al. 2016; Carnethon et al. 2017). Future work should confirm our findings with a large trial that is sufficiently powered to perform stratified analyses among AF and other ethnic/racial groups with hypertension to better inform clinical decision making regarding the use of exercise as antihypertensive lifestyle therapy.

## Conflict of Interest

None declared.
